# Thermal and Morphological Analysis of Linear Low-Density Polyethylene Composites Containing d-limonene/β-cyclodextrin for Active Food Packaging

**DOI:** 10.3390/molecules28031220

**Published:** 2023-01-26

**Authors:** Monika Dobrzyńska-Mizera, Monika Knitter, Marlena Piss, Cristina Del Barone, Salvatore Mallardo, Gabriella Santagata, Maria Laura Di Lorenzo

**Affiliations:** 1 Institute of Materials Technology, Polymer Division, Poznan University of Technology, Piotrowo 3, 61-138 Poznan, Poland; 2 National Research Council (CNR), Institute of Polymers, Composites and Biomaterials (IPCB), c/o Comprensorio Olivetti, via Campi Flegrei, 34, 80078 Pozzuoli, NA, Italy

**Keywords:** linear low-density polyethylene, polymer composite, food packaging

## Abstract

Composites made of linear low-density polyethylene (LLDPE) and β-cyclodextrin/d-limonene inclusion complex (CD-lim) were prepared by melt extrusion to develop a novel food packaging material. Scanning electron microscopy evidenced a fairly good dispersion of the filler within the polymeric matrix. Infrared spectroscopy coupled with thermogravimetric analysis confirmed the presence of CD-lim in the composites, proving that the applied technology of including the essential oil within β-CD cages allows for preventing a sizable loss of d-limonene despite a high temperature and shear applied upon extrusion processing. Moreover, the influence of the filler on the thermal properties of PE was assessed. It was found that the cyclodextrin-based inclusion complex significantly fastens the crystallization path of the polyethylene matrix with an improved crystallization rate of the PE/CD-lim composites compared to the neat polymer.

## 1. Introduction

Packaging materials are commonly based on polyolefins, mostly polyethylene (PE) and polypropylene (PP), due to their excellent processing, optical, barrier, and mechanical properties [1,2]. Almost half of the plastic packaging available on the market is made of polyethylene, which is attainable in a vast variety of molecular structures, with the most common being high-density, low-density, and linear low-density polyethylenes [3,4,5,6]. Linear low-density polyethylene (LLDPE), used in this study, is a semi-crystalline polymer consisting of three different morphological phases, namely, crystalline and amorphous parts linked through an interphase comprising rigid amorphous chain segments [7,8]. Linear low-density polyethylene is most often used to produce reusable and/or single-use bags, trays, agricultural films, and industrial and consumer packaging, including shrink and stretch films, as well as food and specialty containers [9]. LLDPE represents a unique combination of toughness, tear resistance, processability, and low melting temperatures, with the latter especially important in this study, as detailed below. Despite all the beneficial features of polyethylene, there is still a need to improve the material for modern active food packaging designs. 

Food packaging is a common, often neglected product; however, it plays a predominant role in everyday life with the most important aim to provide protection from chemical, biological, and physical alterations. Current trends such as sustainability, environmental impact reduction, and shelf-life extension have gradually become among the most important aspects of designing a packaging system. Active packaging systems are able to reduce food waste by providing, apart from an inert barrier against external agents, several additional functions associated with food preservation, e.g., antimicrobial or antifungal properties [10]. The introduction of natural ingredients acting towards specific pathogens, with an emphasis on their positive impact on health, has been widely discussed in the literature [3,11,12]. Essential oils (EOs) are naturally antimicrobial and antifungal additives currently used as food preservatives and active ingredients in food packaging [11,13,14,15,16,17,18]. These volatile substances may be extracted from several citruses (such as orange, lemon, mandarin, lime, grapefruit, etc.) and/or other plants such as garlic, thymol, oregano, moringa, tea tree, etc. [19]. Unfortunately, their incorporation into polymer matrices may be challenging because the melting point of most thermoplastics exceeds essential oil boiling points. This, in turn, prevents the direct melt mixing of the components and hinders the antimicrobial properties of the modifier. Therefore, the authors proposed an effective route that enabled enhancing the thermal stability of an antimicrobial agent, i.e., the encapsulation of an essential oil, namely, d-limonene, within a cyclodextrin cage-like host (CD-lim inclusion complex). This has been widely discussed in our previous publications [20,21,22]. We proved that the CD-lim content in poly(butylene succinate) (PBS) and poly(lactic acid) (PLLA) matrices is able to extend shelf life and improve product quality by both preventing bacteria and fungi growth and enhancing UV protection. More importantly, the obtained formulations were fully biobased and biodegradable. However, those beneficial features were accompanied by deteriorated mechanical properties, which, especially in the case of naturally stiff PLLA, may be a limiting factor. Taking into account other occurring issues that cannot be affected, e.g., high cost, yellowish color, low transparency, etc., the potential application of these biodegradable and biobased polymers may be difficult, mostly when a long life use time is foreseen for the specific item. Therefore, despite the continuous development of biobased materials and the drive towards eco-friendly solutions, there are applications, especially large-scale industrial ones, where synthetic thermoplastics are still preferred.

It is worth underlining that this issue, despite being significant, is often addressed as controversial, also from an environmental point of view. In fact, biodegradable polymers are often difficult to recycle because their reprocessing at high temperatures in many cases involves material degradation and, in turn, worsened properties. Conversely, like most thermoplastic petroleum-based materials, polyethylene can be easily recycled into other products, which is more cost-effective than manufacturing a new product from virgin plastic. Just to give some context, reusing old plastic saves from 80 to 90% of the energy that would be required to make the same item from virgin materials [23]. On the basis of the previous considerations and with an aim to evaluate the potentiality of polyethylene-based films as novel bioactive food packaging materials, the current research focuses on the production of linear low-density polyethylene-based composites, in which the CD-lim inclusion complex represents the functional dispersed phase.

To the best of our knowledge, such a coordinated system consisting of PE and the CD-lim inclusion complex has not been presented in the literature yet. The idea exploited the outstanding antibacterial properties of encapsulated d-limonene, as proved in our previous publications [20,21,22], which was further dispersed within the linear low-density polyethylene matrix. To our knowledge, only limited literature is available on polyethylene-based composites containing β-CD filled with an active compound: an essential oil, such as carvacrol and cinnamaldehyde [24], or *Ferula asafoetida* leaf and gum extract [25], but no data are available on PE containing the CD-lim inclusion complex.

Thermal, structural, and morphological analysis of the PE/CD-lim composites are detailed and discussed in this manuscript, which is mainly focused on the assessment of the preparation route, including the quantification of essential oil within the polymer and its homogeneous dispersion. As this study aims at the development of a novel material for active food packaging, specific material properties, including optical (haze, gloss, and transparency), barrier, and mechanical (Young’s modulus, elongation at break, and tensile strength), as well as antimicrobial properties, against a variety of gram-positive and negative bacteria and fungi will be presented in a forthcoming manuscript.

## 2. Results and Discussion

**Figure 1:**
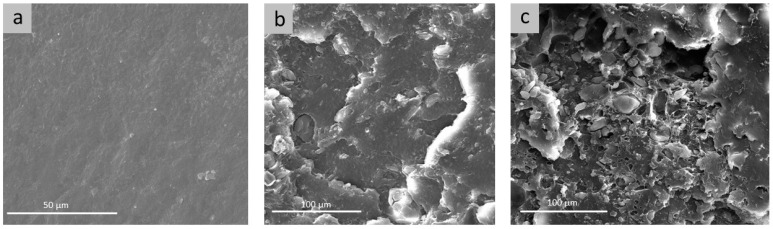
SEM micrographs of PE (**a**), PE/20CD (**b**), and PE/20CD-lim (**c**).

The morphology of PE/CD-lim composites was investigated by scanning electron microscopy. The electron micrographs of cryogenically fractured cross-sections of the composites are reported in Figure 1 and compared with plain PE. For the sake of brevity, only the composites including 20 wt% of CD and 20 wt% of the CD-lim complex were reported and compared. The fractured surface of compression-molded linear low-density polyethylene (Figure 1a) appeared quite smooth, as expected. When 20 wt% of CD was added to the neat polymer (Figure 1b), surface morphology displayed small voids, as well as embedded particles distributed along the whole surface of the sample, with some larger particles also visible on the sample surface. The averaged sizes of the voids and particles were 5–10 μm, wherein some large particles also appeared. Both dispersed particles and empty holes are much larger than β-CD molecules, whose external diameter is 1.53 nm [26].

As far as PE/20CD-lim composites are concerned, it is worth observing the presence of a coarsened fractured surface, with the dark perimeter area corresponding to the hole left in the PE matrix from the discrete domains of the CD-lim complex aggregation detachment and subsequent pulling out. The hole shapes confirmed that an adhesive failure (debonding at the particle/polymer interface) rather than cohesive failure (either in the particle or in the matrix) occurred, in this way strengthening the absence of physical compatibility between linear low-density polyethylene and the CD-lim inclusion complex. These outcomes are widely described in the literature for similar biocomposites, in particular when natural hydrophilic fillers are included in a hydrophobic matrix [27]. Actually, cyclodextrins have hydroxyl groups on the outer surfaces; thus, a poor physical affinity with a hydrophobic matrix such as polyethylene is expected, leading to the aggregation of the β-CD particles. Nevertheless, the gathered filler particles appeared homogeneously distributed within the polyethylene matrix, as seen in Figure 1c. Moreover, only a part of the filler was pulled out during the cryogenic fracture process; the particles rather remained attached to the matrix, despite the expected poor compatibility between the components. Moreover, a plastic cryogenic fracture, characterized by smoothed fracture planes could be observed in the presence of the CD-lim complex. Presumably, during the thermal processing, some limonene, likely adsorbed on the CD surface, could migrate in between the PE macromolecular network, slightly improving the physical interaction between hydrophobic residues and the interfacial adhesion between the polymer and CD particles. This hypothesis was confirmed by spectroscopic analysis, as evidenced in the FTIR-ATR discussion presented below.

FTIR-ATR was performed to assess the presence of the main functional groups of linear low-density polyethylene, β-CD, and limonene inside the PE film after the thermal processing performed to obtain the sheets.

**Figure 2:**
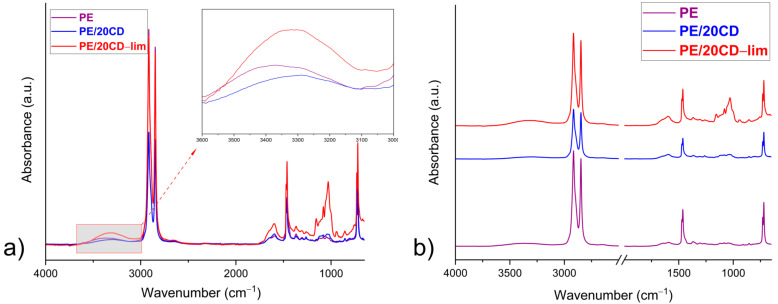
FTIR-ATR of PE (purple curve), PE/20CD (blue curve), and PE/20CD-lim (red curve) for overlapped (**a**) and stacked (**b**) spectra.

For clarity of the discussion of the FTIR-ATR profiles, only neat PE, PE/20CD, and the sheet doped with 20 wt% of CD-lim inclusion complex spectra are reported in Figure 2a,b since the FTIR profile of the linear low-density polyethylene sheet containing 30 wt% of CD-lim shows the same functional groups. As far as PE’s main functional residues are concerned, two strong asymmetric and symmetric stretching vibrations of the –C–H methylene groups were observed at 2915 and 2848 cm^−1^, respectively. The band observed at 1470 cm^−1^ was due to the asymmetric deformation vibrations of the same methylene group, whereas the band occurring around 1462 cm^−1^ was ascribed to CH_2_ out-of-plane wagging mode. The sharp band occurring at around 720 cm^−1^ was attributed to CH_2_ rocking vibrations. Due to PE crystallinity, this peak was split with an additional maximum observed at around 730 cm^−1^.

In addition, weak bands located at 1250 and 1160 cm^−1^, as well as at 1367 and 1135 cm^−1^, appeared due to the asymmetric and symmetric bending vibration of the CH group and to CH_2_ wagging and CH_2_ twisting vibrations, respectively [28]. The FTIR spectra of β-CD and d-limonene have been already shown by the authors in their previous paper [20] and, in order not to overburden the spectra, they are reported in Figure S1a,b of the Supplementary Materials. Nevertheless, their main absorption peaks are detailed as follows: The main characteristic peaks of the cyclic oligosaccharide can be found at around 3300 cm^−1^ due to the stretching vibrations of intra–inter molecular OH hydrogen bonded groups and/or of interstitial water molecules. Moreover, the intense peaks at 2925 and 2854 cm^−1^ due to C–H asymmetric and symmetric stretching modes were also visible. In addition, a peak at around 1650 cm^−1^ concerned the vibration frequencies of H–O–H deformation bands of different types of water molecules located inside β-CD cavities. Finally, the peaks at 1153 cm^−1^ and 1029 cm^−1^ indicated C–O–C and C–H overtone stretching, respectively.

The FTIR-ATR spectrum of d-limonene shows the following characteristic bands: 3074 and 3011 cm^−1^ (=C–H stretching vibrations), 2964 and 2921 cm^−1^ (C–H stretching vibrations), and 1643 and 1676 cm^−1^ (C–C stretching vibrations), respectively, of the ring and vinyl group, as discussed in Ref. [29]. Unfortunately, most of the vibration frequencies typical of β-CD and d-limonene functional groups are covered by the prominent PE absorption peaks, as shown in Figure 2 for overlapped (Figure 2a) and stacked (Figure 2b) spectra. Anyway, two main differences can be highlighted from Figure 2a,b. The first one concerns the successive rise in vibrational absorption modes around 3000–3500 cm^−1^ passing from the neat polymer up to the PE/20CD-lim system. This outcome was likely due to the increase in both β-CD hydroxyl groups and d-limonene vinyl stretching vibrations. The second one is related to the peak rise at around 1600 cm^−1^ due to the vibrational modes of β-CD water molecules and the C–C stretching vibration of the d-limonene vinyl residues.

**Figure 3:**
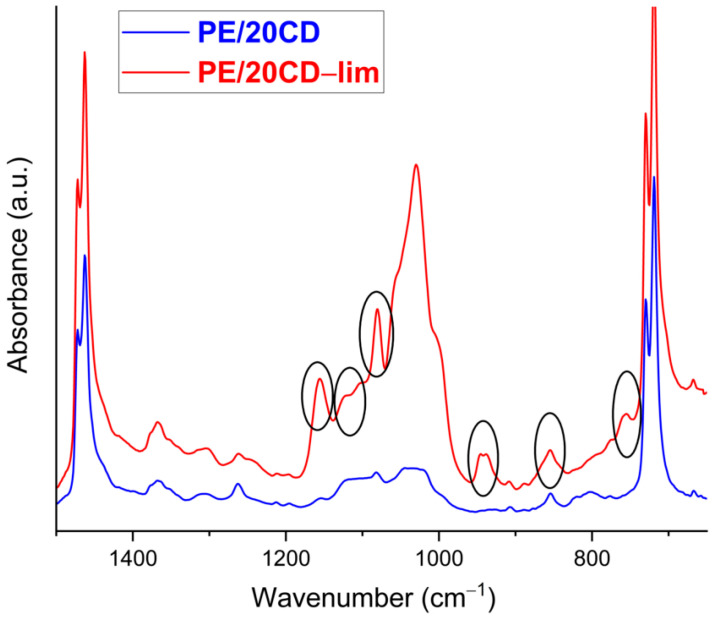
FTIR-ATR spectra of PE/20CD and PE/20CD-lim.

The authors conducted a qualitative approach to verify the presence of β-CD inside linear low-density polyethylene sheet and their likely interaction by means of a spectral subtraction between PE/20CD and PE in a magnified absorbance scale (a multiplying factor of about 0.4). The positive insights are reported and discussed in Figure S2 of the Supplementary Materials. In order to verify the presence of d-limonene inside β-CD torus-shaped cavity sites and, thus, inside the PE polymer matrix, PE/20CD-lim and PE/20CD spectra were overlapped, and the attention was mostly focused in the finger-print region, where the main vibrational modes typical of d-limonene can be found. In Figure 3, the spectra of PE/20CD-lim (red curve) and PE/20CD (blue curve) sheets are reported. It is worthy to observe the presence of peaks (black circles) in the bioactive functionalized film at 1158, 1125, 1080, 945, 855, and 760 cm^−1^ corresponding to the different C–C, C–H bending, and deformations of the ring and vinyl group in and out of plane [30,31,32]. The presence of the above peaks suggests that d-limonene was well encapsulated in β-CD hydrophobic cages and that the whole bioactive complex was entrapped in the PE matrix and preserved also after the extrusion, confirming its high-performant thermal stability. Although the previous outcomes likely suggest the presence of the inclusion complex in the polymer matrix, thermogravimetric analysis was also performed for quantitative confirmation.

**Figure 4:**
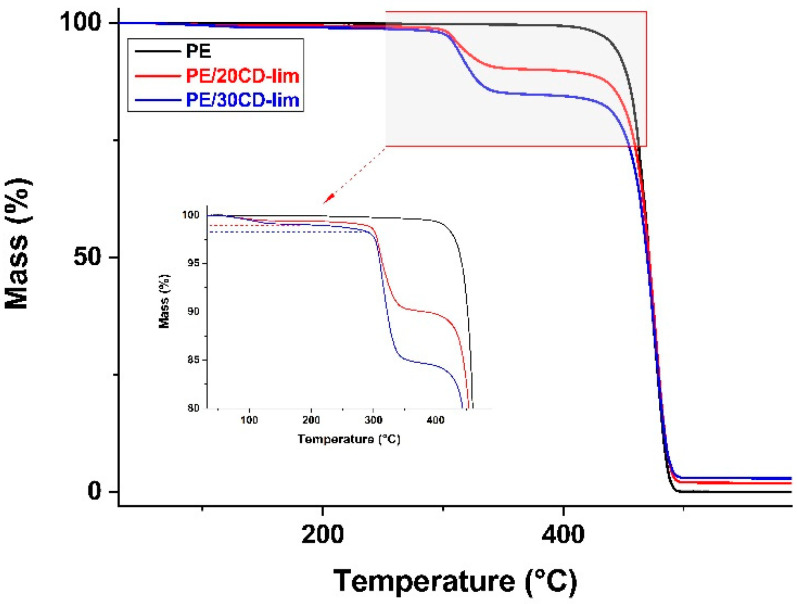
Thermogravimetric analysis (TGA). Mass loss upon heating in nitrogen atmosphere of PE/CD-lim composites compared to pure PE matrix.

Thermogravimetric analysis upon heating of plain PE and PE/CD-lim composites are reported in Figure 4 and Table 1 as mass loss as a function of time, with values normalized with respect to the initial sample mass. Plain polyethylene starts to lose mass around 450 °C upon heating at 10 K min^−1^ in nitrogen inert atmosphere, with a single mass-loss step that is completed before 500 °C and no sizable ash residue remaining, which is in agreement with the literature data [33,34,35,36]. Decomposition is caused by random chain scission followed by the radical transfer process, which is typical for polyolefins [33,37].

The addition of the CD-lim inclusion complex largely varies the pyrolysis profile of polyethylene, with the appearance of multiple degradation steps, highlighted in the enlarged part. The varied profile is caused by the partial evaporation process of d-limonene that overlaps with the release of free and freeze-bound water on the β-CD outer surface up to 120–130 °C [21], followed by a slow mass release when d-limonene is gradually released from β-CD cages, then by a mass drop that starts around 300 °C due to the decomposition of β-cyclodextrin. The mass remaining before the thermal degradation of β-CD allows for estimating the fraction of terpene oil that remains within the PE/CD-lim composites after processing [21]. At 290 °C, the PE/20CD-lim lost 1.1% of the initial mass, whereas the sample containing 30 wt% of filler had a mass decrease of 1.8% compared to the original value, as highlighted by the blue- and red-dotted horizontal lines in the plot. The fraction of d-limonene within the sample, estimated from the enlarged plots, compares well with the expected content of terpene oil in the composites. In fact, the procedure used to prepare the CD-lim inclusion complex allows the inclusion of 7 m% of limonene within β-CD [21]; hence, the theoretical values expected within the composites are 1.4 and 2.1 m% for the PE samples containing 20 and 30 wt% of CD-lim, respectively. These data indicate that only a small fraction of d-limonene is lost during the preparation of the composites and that the preparation route allows for successfully preparing composites containing d-limonene encapsulated within β-cyclodextrin cages.

**Figure 5:**
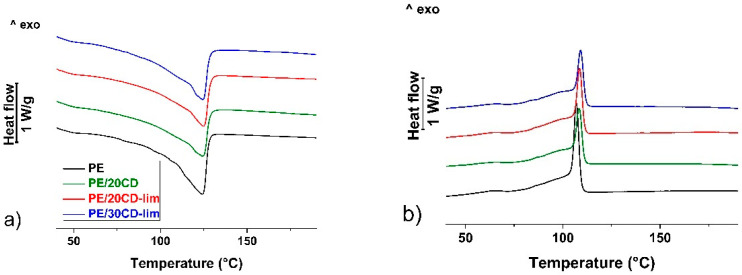
Differential Scanning Calorimetry (DSC). First heating at 10 K min^−1^ (**a**) and subsequent cooling at 5 K min^−1^ (**b**).

Thermal analysis of linear low-density polyethylene, both plain and modified with β-CD or CD-lim, is presented in Figure 5a,b. Figure 5a illustrates the thermal profiles of compression-molded sheets upon heating at 10 K min^−1^. All plots display a major melting endotherm peak at 124.5 °C, whose position does not vary with composition. Conversely, the size of the endotherm is affected by filler content, revealing a varied crystallinity of the material. Crystal fraction was estimated by a comparison of the experimental melting enthalpy with the thermodynamic value taken from [38]. Crystallinity values normalized to PE content (*X*_cr_) are presented in Table 1, which evidences the effect of the natural filler in favoring the crystallization of LLDPE. Compression-molded plain PE displays a crystal fraction of 54%, which increases to 60–64% upon the addition of β-CD or CD-lim.

Further information on the effect of the filler on the crystallization of linear low-density polyethylene was gained by cooling the melted polymers at 5 K min^−1^, with the corresponding heat flow rate plots presented in Figure 5b. The cooling profile of the plain polymer is typical of LLDPE, with a major sharp exotherm followed by a much broader exotherm that extends down to low temperatures [39,40]. The onset of the major exotherm, measured as the intersection of the inflectional tangent with the extrapolated baseline [41], is affected by composition, with the data from the various samples also reported in Table 1. The onset of the crystallization of plain linear low-density polyethylene, occurring at 109.7 °C, is anticipated to be 111.2 °C in the composite containing 20 wt% of β-CD or CD-lim and 112.1 °C in the composite containing 30 wt% of filler. These data evidence that β-CD, either in its emptied form or filled with d-limonene can promote the crystallization of LLDPE in its early stage, probably favoring crystal nucleation. A similar nucleating effect was also reported for poly(l-lactic acid) composites containing β-CD containing d-limonene [21], which suggests that CD-lim may also be used as an additive to enhance the crystallization rate.

## 3. Conclusions

Linear low-density polyethylene composites containing the β-cyclodextrin/d-limonene inclusion complex were prepared via melt mixing in various formulations, from 20 to 30 wt% of filler content, and their structural, thermal, and morphological properties were presented and discussed here. The preparation route via extrusion mixing ensures the homogeneous distribution of β-CD and CD-lim particles within the polyethylene matrix, as proved by scanning electron microscopy analysis. The homogeneous dispersion of β-cyclodextrin within polyethylene was not a priori expected, because cyclodextrins have hydroxyl groups on the outer surfaces; hence, the poor affinity with a hydrophobic polymer matrix such as polyethylene could have led to the aggregation of the β-CD particles. As shown by infrared spectroscopy analysis, it is likely that during the melt processing, part of the limonene, possibly adsorbed on the β-CD surface, could have migrated to PE, thus, improving the interaction and interfacial adhesion between the polymer and the filler particles.

Most importantly, the inclusion of d-limonene within β-CD cavities prevents the loss of the volatile essential oil upon composite preparation. Despite the fact that the high processing temperature should lead to large evaporation of the terpene oil, a sizable amount of d-limonene was proven to remain within the composites. The inclusion of d-limonene within the β-CD cages avoids the release of the volatile compound upon melt extrusion, as only a small fraction of the terpene is lost during the preparation of the composites. Therefore, the preparation route detailed here allows for successfully preparing composites containing d-limonene encapsulated within β-cyclodextrin cages, which is a key issue for the antibacterial properties of the films.

Moreover, the fillers act as nucleating agents for polyethylene, as the films containing 20–30 wt% of modifier have increased crystallization temperature from melt and enhanced crystallinity degree compared to neat polyethylene. The enhanced crystal fraction is of importance to evaluate the influence of the filler on material properties. In fact, the addition of the CD-lim modifier into the linear low-density polyethylene matrix is also expected to influence other—crucial from a food packaging perspective—properties, such as optical, barrier, and mechanical features. Therefore, an analysis of the above will be presented and discussed in a forthcoming paper, together with a thorough investigation of the antibacterial and antifungal activity of this novel material.

## 4. Methodology

### 4.1. Materials

A commercial linear low-density polyethylene Dowlex^TM^ 2045G, abbreviated PE or LLDPE, with MFR 1 g/10 min (190 °C, 2.16 kg), and 1-octene comonomer content of 2.7 mol% was purchased from The Dow Chemical Company (Midland, MI, USA) [42]. β-cyclodextrin (β-CD) with a purity of ≥99% was provided by Cyclodextrin Shop (Tilburg, the Netherlands). The materials were used after drying under vacuum at 50 °C for 24 h before extrusion. d-limonene technical grade (~90% purity) was supplied by Sigma-Aldrich and used as a raw material in the microencapsulation process.

### 4.2. Preparation of CD-lim Inclusion Complex and Composites

An inclusion complex of β-cyclodextrin and d-limonene was obtained via precipitation, as detailed in our previous works [20,21,22]. PE pellets were mixed with CD-lim complex in a rotary mixer Retsch, type GM 200 for 3 min at a rotation speed of 2000 rpm. Homogenization of the premixed materials with different CD-lim inclusion complex contents (from 0 to 30 wt%) was ensured by molten state extrusion with a Zamak corotating twin-screw extruder operated at 160 °C and 60 rpm. The extruded rod was cooled in air and pelletized. PE-based composites modified with CD-lim at various compositions were prepared, as summarized in Table 2.

The composites were prepared via the compression molding method using a hydraulic press, manufactured by the Remi-Plast (Czerwonak, Poland), at a temperature of 160 °C at a maximum load of about 180 Pa to obtain 2 mm thick sheets. Prior to the forming process, the materials were dried under vacuum at 50 °C for 24 h. The samples were cooled in the air at room temperature after thermal processing.

### 4.3. Methods

#### 4.3.1. Scanning Electron Microscopy (SEM)

Cryogenically fractured cross-sections of PE-based composites were analyzed using a Quanta 200 FEG, 338 FEI scanning electron microscope (Thermo Fisher Scientific, Eindhoven, the Netherlands). SEM microphotographs were collected at room temperature and voltage of 20 kV. Before analysis, the surfaces of the samples were sputter-coated with an 18 ± 0.2 nm layer of Au-Pd alloy by a MED 020 splattering device, Bal-Tec AG (Pfaffikon, Switzerland).

#### 4.3.2. Attenuated Total Reflection Fourier-Transform Infrared (FTIR-ATR)

Attenuated total reflection fourier-transform infrared (FTIR-ATR) spectroscopy of PE/CD-lim composites was carried out on the surface of the compression-molded films. Details of the FTIR-ATR spectra of neat β-CD, d-limonene, and CD-lim complex were performed and reported in [20]. The spectra were collected on a PerkinElmer Spectrum 100 spectrometer equipped with a Universal ATR diamond crystal sampling accessory (Netzsch, Waltham, MA, USA). All the samples were analyzed at room temperature in the range of 4000–480 cm^−1^, recorded as an average of 64 scans with a resolution of 4 cm^−1^. Before testing, all samples were dried in an oven at 60 °C for 24 h.

#### 4.3.3. Differential Scanning Calorimetry (DSC)

Thermal properties were investigated with a Netzsch DSC 204 F1 Phoenix^®^ (Netzsch, Selb, Germany) apparatus, using aluminum crucibles and 3 ± 0.5 mg samples under nitrogen flow. High purity standards were used to calibrate the instrument, including indium, tin, bismuth, zinc, and aluminum. Indium melting enthalpy was used for energy calibration. All the samples were heated from 30 °C to 200 °C at a heating rate of 10 °C min^−1^ and held in a molten state for 5 min and then cooled to 30 °C at 5 °C min^−1^.

#### 4.3.4. Thermogravimetric analysis (TGA)

TGA analyses were performed in the temperature range between 30 and 800 °C, at a heating rate of 10 °C min^−1^, under a nitrogen atmosphere using a Netzsch TG 209 F1 apparatus (Netzsch, Selb, Germany) calibrated by analyzing several standards, including In, Sn, Bi, Zn, Al, and Ag. The decomposition onset temperature, *T*_o_, of approximately 10 mg samples, was determined at the intersection of tangents to two branches of the thermogravimetric curve [43]. Each measurement was preceded by an empty pan run, which was subtracted from each thermogram to correct instrumental drift [44].

## Figures and Tables

**Table 1:** Crystallinity degree, crystallization onset temperature (*T*_c,ons_), degradation onset temperature (*T*_d,ons_), and d-limonene content in the PE/CD-lim composites.

Sample Name	DSC		TGA	
	*X*_cr_ (%)	*T*_c,ons_ (°C)	*T*_d,ons_ (°C)	d-limonene Content (%)
PE	54	109.7	463.5	-
PE/20CD	64	111.2	308.5	-
PE/20CD-lim	63	111.2	302.5	1.1
PE/30CD-lim	60	112.1	303.6	1.8

**Table 2:** Symbols and mass concentrations of samples.

Sample Name	Mass Concentration [wt%]		
	LLDPE	CD	CD-lim
PE	100	0	0
PE/20CD	80	20	0
PE/20CD-lim	80	0	20
PE/30CD-lim	70	0	30

CD: β-cyclodextrin, CD-lim: β-cyclodextrin/d-limonene inclusion complex, and LLDPE: linear low-density polyethylene.

## Data Availability

Not applicable.

## Supplementary Materials

The following supporting information can be downloaded at: https://www.mdpi.com/article/10.3390/molecules28031220/s1, Figure S1: FTIR-ATR spectra of β-CD (a) and D-Limonene (b). Figure S2. FTIR-ATR spectral subtraction in magnified absorbance scale (azure curve) between PE/CD (blue curve) and PE (red curve).

## References

[1] Peres A.M., Pires R.R., Oréfice R.L. (2016). Evaluation of the Effect of Reprocessing on the Structure and Properties of Low Density Polyethylene/Thermoplastic Starch Blends. Carbohydr. Polym..

[2] LaChance A.M., Hou Z., Farooqui M.M., Carr S.A., Serrano J.M., Odendahl C.E., Hurley M.E., Morrison T.E., Kubachka J.L., Samuels N.T. (2022). Polyolefin Films with Outstanding Barrier Properties Based on One-Step Coassembled Nanocoatings. Adv. Compos. Hybrid. Mater..

[3] Zhong Y., Godwin P., Jin Y., Xiao H. (2020). Biodegradable Polymers and Green-Based Antimicrobial Packaging Materials: A Mini-Review. Advanced Industrial and Engineering Polymer Research.

[4] Sperati C.A., Franta W.A., Starkweather H.W. (1953). The Molecular Structure of Polyethylene. V. The Effect of Chain Branching and Molecular Weight on Physical Properties ^1^. J. Am. Chem. Soc..

[5] Garcia-Garcia D., Quiles-Carrillo L., Balart R., Torres-Giner S., Arrieta M.P. (2022). Innovative Solutions and Challenges to Increase the Use of Poly(3-Hydroxybutyrate) in Food Packaging and Disposables. Eur. Polym. J..

[6] El-Wakil A.E.-A.A., Moustafa H., Youssef A.M. (2022). Antimicrobial Low-Density Polyethylene/Low-Density Polyethylene-Grafted Acrylic Acid Biocomposites Based on Rice Bran with Tea Tree Oil for Food Packaging Applications. J. Thermoplast. Compos. Mater..

[7] Kolgjini B., Schoukens G., Kiekens P. (2011). Three-Phase Characterization of Uniaxially Stretched Linear Low-Density Polyethylene. Int. J. Polym. Sci..

[8] Sajkiewicz P., Hashimoto T., Saijo K., Gradys A. (2005). ‘Intermediate Phase’ in Poly(Ethylene) as Elucidated by the WAXS. Analysis of Crystallization Kinetics. Polymer.

[9] Masayuki Y. (1998). Effect of Molecular Structure in Branched Polyethylene on Adhesion Properties with Polypropylene. J. Polym. Sci..

[10] European Food Safety Authority (EFSA) (2009). Guidelines on submission of a dossier for safety evaluation by the EFSA of active or intelligent substances present in active and intelligent materials and articles intended to come into contact with food. EFSA J..

[11] Mousavi Khaneghah A., Hashemi S.M.B., Limbo S. (2018). Antimicrobial Agents and Packaging Systems in Antimicrobial Active Food Packaging: An Overview of Approaches and Interactions. Food Bioprod. Process..

[12] Moeini A., van Reenen A., van Otterlo W., Cimmino A., Masi M., Lavermicocca P., Valerio F., Immirzi B., Santagata G., Malinconico M. (2020). α-Costic Acid, a Plant Sesquiterpenoid from Dittrichia Viscosa, as Modifier of Poly (Lactic Acid) Properties: A Novel Exploitation of the Autochthone Biomass Metabolite for a Wholly Biodegradable System. Ind. Crops Prod..

[13] Falleh H., ben Jemaa M., Saada M., Ksouri R. (2020). Essential Oils: A Promising Eco-Friendly Food Preservative. Food Chem..

[14] Huang T., Qian Y., Wei J., Zhou C. (2019). Polymeric Antimicrobial Food Packaging and Its Applications. Polymers.

[15] Tariq S., Wani S., Rasool W., Shafi K., Bhat M.A., Prabhakar A., Shalla A.H., Rather M.A. (2019). A Comprehensive Review of the Antibacterial, Antifungal and Antiviral Potential of Essential Oils and Their Chemical Constituents against Drug-Resistant Microbial Pathogens. Microb. Pathog..

[16] Burt S. (2004). Essential Oils: Their Antibacterial Properties and Potential Applications in Foods—A Review. Int. J. Food Microbiol..

[17] Atta O.M., Manan S., Ul-Islam M., Ahmed A.A.Q., Ullah M.W., Yang G. (2022). Development and Characterization of Plant Oil-Incorporated Carboxymethyl Cellulose/Bacterial Cellulose/Glycerol-Based Antimicrobial Edible Films for Food Packaging Applications. Adv. Compos. Hybrid Mater..

[18] Boro U., Priyadarsini A., Moholkar V.S. (2022). Synthesis and Characterization of Poly(Lactic Acid)/Clove Essential Oil/Alkali-Treated Halloysite Nanotubes Composite Films for Food Packaging Applications. Int. J. Biol. Macromol..

[19] Arrieta M.P., López J., Ferrándiz S., Peltzer M.A. (2013). Characterization of PLA-Limonene Blends for Food Packaging Applications. Polym. Test..

[20] Mallardo S., de Vito V., Malinconico M., Volpe M.G., Santagata G., Di Lorenzo M.L. (2016). Poly(Butylene Succinate)-Based Composites Containing β-Cyclodextrin/d-Limonene Inclusion Complex. Eur. Polym. J..

[21] Dobrzyńska-Mizera M., Knitter M., Mallardo S., Del Barone M.C., Santagata G., Di Lorenzo M.L. (2021). Thermal and Thermo-Mechanical Properties of Poly(L-Lactic Acid) Biocomposites Containing β-Cyclodextrin/d-Limonene Inclusion Complex. Materials.

[22] Dobrzyńska-Mizera M., Knitter M., Szymanowska D., Mallardo S., Santagata G., Di Lorenzo M.L. (2022). Optical, Mechanical, and Antimicrobial Properties of Bio-based Composites of Poly(L-lactic Acid) and D-limonene/Β-cyclodextrin Inclusion Complex. J. Appl. Polym. Sci..

[23] Martín-Lara M.A., Moreno J.A., Garcia-Garcia G., Arjandas S., Calero M. (2022). Life Cycle Assessment of Mechanical Recycling of Post-Consumer Polyethylene Flexible Films Based on a Real Case in Spain. J. Clean Prod..

[24] Canales D., Montoille L., Rivas L.M., Ortiz J.A., Yañez-S M., Rabagliati F.M., Ulloa M.T., Alvarez E., Zapata P.A. (2019). Fungicides Films of Low-Density Polyethylene (LDPE)/Inclusion Complexes (Carvacrol and Cinnamaldehyde) Against Botrytis Cinerea. Coatings.

[25] Niazmand R., Razavizadeh B.M. (2021). Active Polyethylene Films Incorporated with β-Cyclodextrin/Ferula Asafoetida Extract Inclusion Complexes: Sustained Release of Bioactive Agents. Polym. Test..

[26] Szejtli J. (1998). Introduction and General Overview of Cyclodextrin Chemistry. Chem. Rev..

[27] Huber T., Müssig J. (2008). Fibre Matrix Adhesion of Natural Fibres Cotton, Flax and Hemp in Polymeric Matrices Analyzed with the Single Fibre Fragmentation Test. Compos. Interfaces.

[28] Bredács M., Barretta C., Castillon L.F., Frank A., Oreski G., Pinter G., Gergely S. (2021). Prediction of Polyethylene Density from FTIR and Raman Spectroscopy Using Multivariate Data Analysis. Polym. Test..

[29] Partal Ureña F., Moreno J.R.A., López González J.J. (2009). Conformational Study of (R)-(+)-Limonene in the Liquid Phase Using Vibrational Spectroscopy (IR, Raman, and VCD) and DFT Calculations. Tetrahedron Asymmetry.

[30] Marcuzzo E., Debeaufort F., Sensidoni A., Tat L., Beney L., Hambleton A., Peressini D., Voilley A. (2012). Release Behavior and Stability of Encapsulated <scp>d</Scp> -Limonene from Emulsion-Based Edible Films. J. Agric. Food Chem..

[31] Cava D., Catala R., Gavara R., Lagaron J.M. (2005). Testing Limonene Diffusion through Food Contact Polyethylene by FT-IR Spectroscopy: Film Thickness, Permeant Concentration and Outer Medium Effects. Polym. Test..

[32] Derdar H., Belbachir M., Harrane A. (2019). A Green Synthesis of Polylimonene Using Maghnite-H+, an Exchanged Montmorillonite Clay, as Eco-Catalyst. Bulletin of Chemical Reaction Engineering & Catalysis.

[33] Zhao D., Wang X., Miller J.B., Huber G.W. (2020). The Chemistry and Kinetics of Polyethylene Pyrolysis: A Process to Produce Fuels and Chemicals. ChemSusChem.

[34] Dubdub I., Al-Yaari M. (2020). Pyrolysis of Low Density Polyethylene: Kinetic Study Using TGA Data and ANN Prediction. Polymers.

[35] Aboulkas A., el harfi K., el Bouadili A. (2010). Thermal Degradation Behaviors of Polyethylene and Polypropylene. Part I: Pyrolysis Kinetics and Mechanisms. Energy Convers. Manag..

[36] Kaci M., Cimmino S., di Lorenzo M.L., Silvestre C., Sadoun T. (1999). Effect of The Thermo-Oxidation and Natural Weather on The Structure, Morphology, and Properties of Unstabilized and Hals-Stabilized LDPE Films. J. Macromol. Sci. Part A.

[37] Das P., Tiwari P. (2017). Thermal Degradation Kinetics of Plastics and Model Selection. Thermochim. Acta.

[38] Niu B., Chen J.-B., Chen J., Ji X., Zhong G.-J., Li Z.-M. (2016). Crystallization of Linear Low Density Polyethylene on an in Situ Oriented Isotactic Polypropylene Substrate Manipulated by an Extensional Flow Field. CrystEngComm.

[39] Arndt J.-H., Brüll R., Macko T., Garg P., Tacx J.C.J.F. (2018). Characterization of the Chemical Composition Distribution of Polyolefin Plastomers/Elastomers (Ethylene/1-Octene Copolymers) and Comparison to Theoretical Predictions. Polymer.

[40] Androsch R. (1999). Melting and Crystallization of Poly(Ethylene-Co-Octene) Measured by Modulated d.s.c. and Temperature-Resolved X-Ray Diffraction. Polymer.

[41] Wunderlich B. (1990). Thermal Analysis.

[42] Hsu Y.-C., Truss R.W., Laycock B., Weir M.P., Nicholson T.M., Garvey C.J., Halley P.J. (2017). The Effect of Comonomer Concentration and Distribution on the Photo-Oxidative Degradation of Linear Low Density Polyethylene Films. Polymer.

[43] Dobrzyńska-Mizera M., Knitter M., Woźniak-Braszak A., Baranowski M., Sterzyński T., Di Lorenzo M.L. (2020). Poly(l-Lactic Acid)/Pine Wood Bio-Based Composites. Materials.

[44] Vyazovkin S., Chrissafis K., Di Lorenzo M.L., Koga N., Pijolat M., Roduit B., Sbirrazzuoli N., Suñol J.J. (2014). ICTAC Kinetics Committee Recommendations for Collecting Experimental Thermal Analysis Data for Kinetic Computations. Thermochim. Acta.

