# Supplementary material for: Thermal and Morphological Analysis of Linear Low-Density Polyethylene Composites Containing d-limonene/β-cyclodextrin for Active Food Packaging
Source: Molecules. 2023 Jan 26;28(3):1220. doi: 10.3390/molecules28031220 (PMC9920889; doi:10.3390/molecules28031220)
Supplement: Supplementary file 1 [file molecules-28-01220-s001.zip › molecules-2142700-supplementary.pdf]

molecules

Thermal and morphological analysis of linear low-density polyethylene composites containing D-limonene/β-cyclodextrin for active food packaging

Monika Dobrzyńska-Mizera [a]*, Monika Knitter [a], Marlena Piss [a], Cristina Del Barone [b], Salvatore Mallardo [b], Gabriella Santagata [b] and Maria Laura Di Lorenzo [b]

1. Methodology

1.1. Attenuated Total Reflection Fourier-Transform Infrared (FTIR-ATR)

Attenuated total reflection Fourier-transform infrared (FTIR-ATR) spectroscopy of PE/CD-lim composites was carried out on the surface of the compression-molded sheets. Details of the FTIR-ATR spectra of neat β-CD, D-limonene, and the CD-lim complex are reported in [20]. The spectra were collected on a PerkinElmer Spectrum 100 spectrometer, equipped with a Universal ATR diamond crystal sampling accessory. All the samples were analyzed at room temperature in the range of 4000–480 cm^{-1}, recorded as an average of 64 scans with a resolution of 4 cm^{-1}. Before testing, all samples were dried in an oven at 50 °C for 24 h.

2. Results and Discussion

The main characteristic peaks of β-CD (Figure S1a) can be found at around 3300 cm^{-1} due to the stretching vibrations of intra–inter molecular OH hydrogen-bonded groups and/or of interstitial water molecules. Moreover, intense peaks at 2925 and 2854 cm^{-1} due to C–H asymmetric and symmetric stretching modes were also visible. In addition, a peak at around 1650 cm^{-1} concerned the vibration frequencies of the H–O–H deformation bands of different types of water molecules located inside β-CD cavities. Finally, the peaks at 1153 cm^{-1} and 1029 cm^{-1} indicated C–O–C and C–H overtone stretching, respectively.

FTIR-ATR spectrum of D-limonene (Figure S1b) shows the following characteristic bands: 3074 and 3011 cm^{-1} (=C–H stretching vibrations), 2964 and 2921 cm^{-1} (C–H stretching vibrations), and 1643 and 1676 cm^{-1} (C–C stretching vibrations) of the ring and the vinyl group, as discussed in Ref. [27]. Unfortunately, most of the vibration frequencies typical of β-CD and D-limonene functional groups are covered up by the prominent PE absorption peaks, as shown in Figure S2.

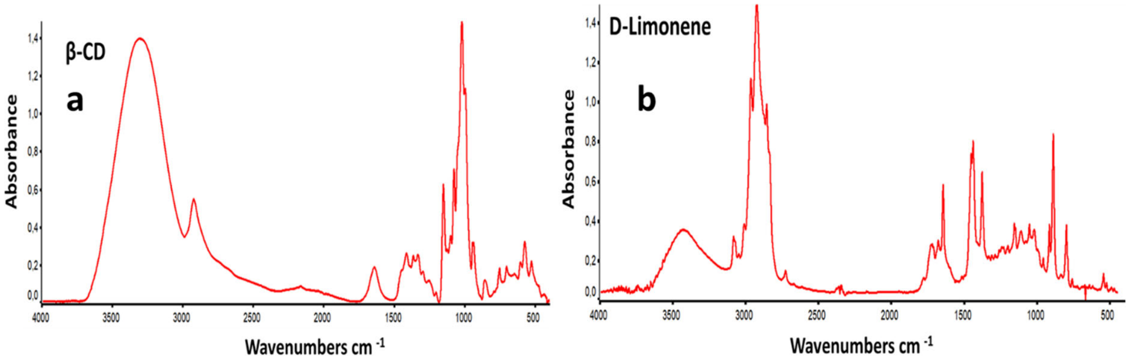

Figure S1. FTIR-ATR spectra of β-CD (**a**) and D-Limonene (**b**).

To verify the presence of β-CD inside the PE film and their likely interaction, spectral subtraction between PE/20CD and PE was performed and reported in Figure S2 in a

magnified absorbance scale (multiplying factor of about 0.4). The spectral subtraction (azure curve) recorded in the region of C-H stretching vibration evidenced two main results; firstly, the PE peak of –C–H methylene groups at 2915 cm^{-1} disappeared in favor of the occurrence of two weak shoulders at about 2930 and 2854 cm^{-1}, which is related to β-CD asymmetric and symmetric vibrational frequencies. This outcome likely confirms the presence of β-CD inside the PE matrix (red circle). Moreover, from the azure curve, it was well observable that the PE peak at 2848 cm^{-1} shifted to lower frequencies, precisely at 2838 cm^{-1}, thus, suggesting the occurrence of some physical interaction between PE and β-CD molecules (black circle).

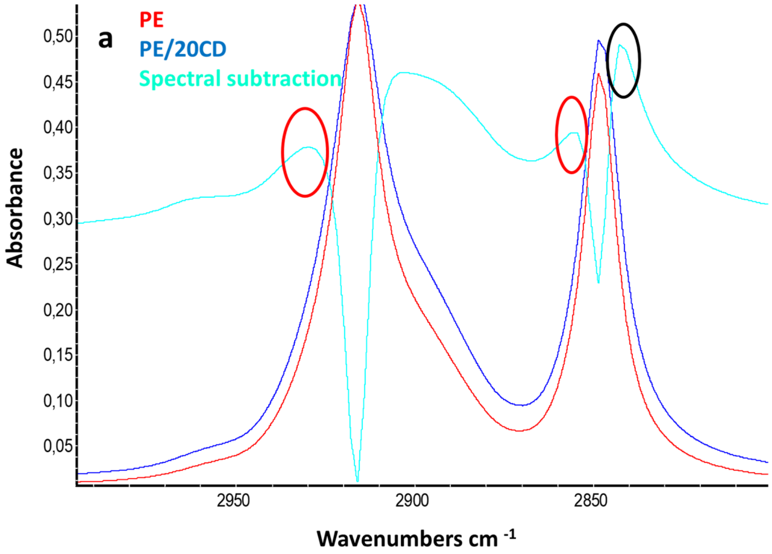

Figure S2. FTIR-ATR spectral subtraction in magnified absorbance scale (azure curve) between PE/CD (blue curve) and PE (red curve).
